# Cerebral hemodynamic response during a live action-observation and action-execution task: A fNIRS study

**DOI:** 10.1371/journal.pone.0253788

**Published:** 2021-08-13

**Authors:** Helga O. Miguel, Emma E. Condy, Thien Nguyen, Selin Zeytinoglu, Emily Blick, Kimberly Bress, Kosar Khaksari, Hadis Dashtestani, John Millerhagen, Sheida Shahmohammadi, Nathan A. Fox, Amir Gandjbakhche

**Affiliations:** 1 National Institute of Child Health and Human Development, National Institutes of Health, Bethesda, Maryland, United States of America; 2 Department of Human Development and Quantitative Methodology, University of Maryland, College Park, Maryland, United States of America; Preeminent Medical Phonics Education & Research Center, Hamamatsu University School of Medicine, JAPAN

## Abstract

Although many studies have examined the location of the action observation network (AON) in human adults, the shared neural correlates of action-observation and action-execution are still unclear partially due to lack of ecologically valid neuroimaging measures. In this study, we aim to demonstrate the feasibility of using functional near infrared spectroscopy (fNIRS) to measure the neural correlates of action-observation and action execution regions during a live task. Thirty adults reached for objects or observed an experimenter reaching for objects while their cerebral hemodynamic responses including oxy-hemoglobin (HbO) and deoxy-hemoglobin (HbR) were recorded in the sensorimotor and parietal regions. Our results indicated that the parietal regions, including bilateral superior parietal lobule (SPL), bilateral inferior parietal lobule (IPL), right supra-marginal region (SMG) and right angular gyrus (AG) share neural activity during action-observation and action-execution. Our findings confirm the applicability of fNIRS for the study of the AON and lay the foundation for future work with developmental and clinical populations.

## Introduction

Recognizing actions performed by others is important for basic social and communicative behavior. The ability to recognize and understand the meaning from others’ actions requires the integration of both perceptive and processing skills [1, 2]. It has been hypothesized that this ability is mediated by a network of brain regions, referred to as the Mirror Neuron System (MNS) that are active when individual perform an action as well as when they observe someone else perform the same action. Additionally, some have gone far as to propose the MNS is implicated in action understanding; however, this theory has been critiqued as human studies have yet to fully support these claims [3].

Brain regions that are activated during observation and execution of the same action include the inferior parietal lobe (IPL), ventral premotor (vPMC) and inferior frontal gyrus (IFG) [4]. These regions are often described as part of a broader network referred to as the action-observation network (AON) that includes also occipitotemporal regions involved in processing of visual information and action [5, 6]. The overlapping neural regions that are active during both observation and execution of an action in humans led researchers to name it the MNS as the homologue of “mirror neurons” originally found in non-human primates using single cell recordings [7, 8]. However, in contrast with animal models, most studies examining MNS in humans rely on non-invasive methods as functional magnetic resonance imaging (fMRI) or electroencephalogram (EEG). There is only one study reported that successfully conducted single-cell recording in humans [9]. This generated a criticism of the “mirror neuron” nomenclature in humans studies [10] but researchers have continued to interpret the regional activation patterns across action-observation and action-execution as support for a neural mirroring or mirror system. AON shares neural correlates with the MNS in frontal-parietal regions [11], therefore, we will frame the present paper as an investigation of the action observation and action execution as two separate networks in light of the AON.

The AON has been studied in humans using electroencephalography (EEG), namely through *mu desynchronization* [12], an event-related desynchronization in the 8–12 Hz frequency range at central sites. Mu desynchronization occurs both when someone performs an action and when they observe someone perform the same action and thus being the predominant measure of AON in the human brain [13]. However, whether mu desynchronization truly reflects a mirroring system in the human brain remains to be controversial [14, 15]. Critiques include the shared frequency band with alpha rhythm [16] or the shared topography with beta rhythm [17, 18] (see [14, 15] for a comprehensive review on the limitations of EEG findings). The lack of spatial resolution provided by EEG has been addressed through studies using functional magnetic resonance imaging (fMRI) [5, 6], which locate more precise regions of brain activity related to the AON. These neuroimaging studies have identified a key set of regions that demonstrate coordinated activation during both observation and execution, including the ventral premotor cortex (PMv, BA6), inferior frontal gyrus (IFG, BA44), and inferior parietal lobe (IPL, BA40) [1, 19]. However, despite its superior spatial resolution, fMRI sensitivity to motion artifact may pose an important limitation to studying AON. This is especially relevant in the study of AON, where the research question requires the direct contrast between observation and execution of an action [13, 20]. Although there are fMRI studies that include an execution condition [21–23] the actions selected might not be ecologically valid given the physical constraints placed on individuals for successful fMRI data collection [6, 24, 25].

Functional near-infrared spectroscopy (fNIRS) is an optical imaging tool that has gained the attention of the AON research community in recent years [26] Like fMRI, fNIRS is a brain imaging technique that measures changes in the oxygenation level of cerebral tissue as a proxy for brain activation. However, rather than relying on the use of large magnets, fNIRS uses near infra-red light to quantify the hemodynamic responses associated with neural activity by measuring changes in oxy-hemoglobin (HbO), deoxy-hemoglobin (HbR) and total hemoglobin (HbT). The increased blood flow that is evoked by neural activity in a brain region is characterized by an increase in HbO and decrease in HbR [27]. Additionally, fNIRS is more tolerant to motion and allows for imaging of brain activity in a wider range of environments, as compared to fMRI [28] and EEG, which makes it a well-suited measure of brain activity for paradigms that use motor actions, as the ones used in the context of AON. fNIRS has several additional advantages compared to other modalities including non-invasiveness, affordability, and portability [27].

fNIRS has been used to examine the AON mostly in the adult population [29–36] and has identified a network of regions consistent with fMRI literature, namely the IFG, vPMC, and IPL [29, 31–34]. The results also implicate other brain regions including the supplementary motor region (SMA) [37] or superior/middle temporal gyri [30]. Recent studies with developing population have found consisting cortical activation patterns [38, 39]. When comparing execution and observation, studies have reported that observing someone perform an action seems to elicit more bilateral activation than executing the same action in the superior/middle temporal gyri, STS, vPMC and somatosensory region [30, 31]. Another consistent finding across studies is that the execution condition elicits a greater hemodynamic response [29, 31] and more specific to motor regions, including the PMC and primary-motor regions [29, 37, 40]. Moreover, executing an action consistently activates premotor regions, whereas observation does not activate these regions [41].

Although these studies have provided information about regions that are active either during the execution or observation of an action, many do not provide contrasts between the conditions to determine which regions overlap between the conditions. Some studies have conducted contrasts between conditions but most have used a unilateral probe [29, 32, 34] limiting the conclusions regarding lateralization differences. Others used a bilateral probe [30], but did not cover all the ROIs hypothesized as candidates of AON. For those that do cover all these regions, contrasts between the conditions are often not reported. For example, Zhang and colleagues found differences in the IFG, dorsolateral prefrontal cortex (dlPFC), PMC, SMA and sensorimotor regions when participants observed others walking compared to when they walked themselves [36] however, they did not report follow up contrasts, and thus conclusions on differences in activation patterns between observation and execution cannot be drawn. While the results from the AON fNIRS literature are not conclusive and need further examination, the parietal cortex, namely the IPL, seems to be identified in several studies during both observation and execution conditions [29, 30, 32–34] and could be a key region of the AON.

In the current study, we use fNIRS to examine brain activity during a live, action-observation paradigm in a sample of healthy adults. The purpose of this study was to properly assess cortical regions associated with the AON in adults and to establish a method to eventually be used in future research investigating the AON in developing populations (infants) and clinical populations who present deficits in action recognition, namely autism spectrum disorders (ASD) [42]. We implement a paradigm that mimics a simple everyday task (reaching and grasping a cup and observing someone reaching and grasping for a cup) while examining specific regions of interest, namely the bilateral sensorimotor and parietal regions (superior parietal lobule and inferior parietal lobule). Although fNIRS has the capability to record brain activation in more ecologically valid situations or during true interactive settings, we decided to replicate a paradigm used in the mirror neuron literature with infant populations using EEG [43]. Further, due to limited number of channels we chose to investigate the sensorimotor cortex as a primary region that activates following execution of actions [11, 44] and the parietal region as a region that has been consistently identified as an AON area [11, 45, 46]. The aim of this study is to demonstrate that fNIRS can be applied to the study of AON during a validated live paradigm. We hypothesize that the live action paradigm will recruit the parietal region, namely the IPL, during both observation and execution of an action as measured by fNIRS. In addition, we hypothesize that sensorimotor regions will be recruited to a greater extent in the execution condition.

## Methods

### Participants

This study included 30 participants (11 males) with a mean age of 33.77 ± 13.60 years old, (range 19–65). Participants had no history of concussion within the previous 12 months. Twenty-four participants were right-handed and 6 were ambidextrous as per the Edinburg Handedness Inventory [47] An additional 5 participants were tested but excluded due to noisy data (*n* = 2), experimental error (*n* = 2) or not having the minimum number of good trials (*n* = 1).

Participants were recruited from the healthy volunteers’ database of the National Institutes of Health, as well as through fliers distributed in the community. Participants were enrolled in this study through the National Institute of Child Health and Human Development’s institutional recruitment procedures. All participants signed an informed consent prior to the start of the experiment and the experimental protocol was approved by the Institutional Review Board.

### Measures

#### NIRS recording

**Fig 1:**
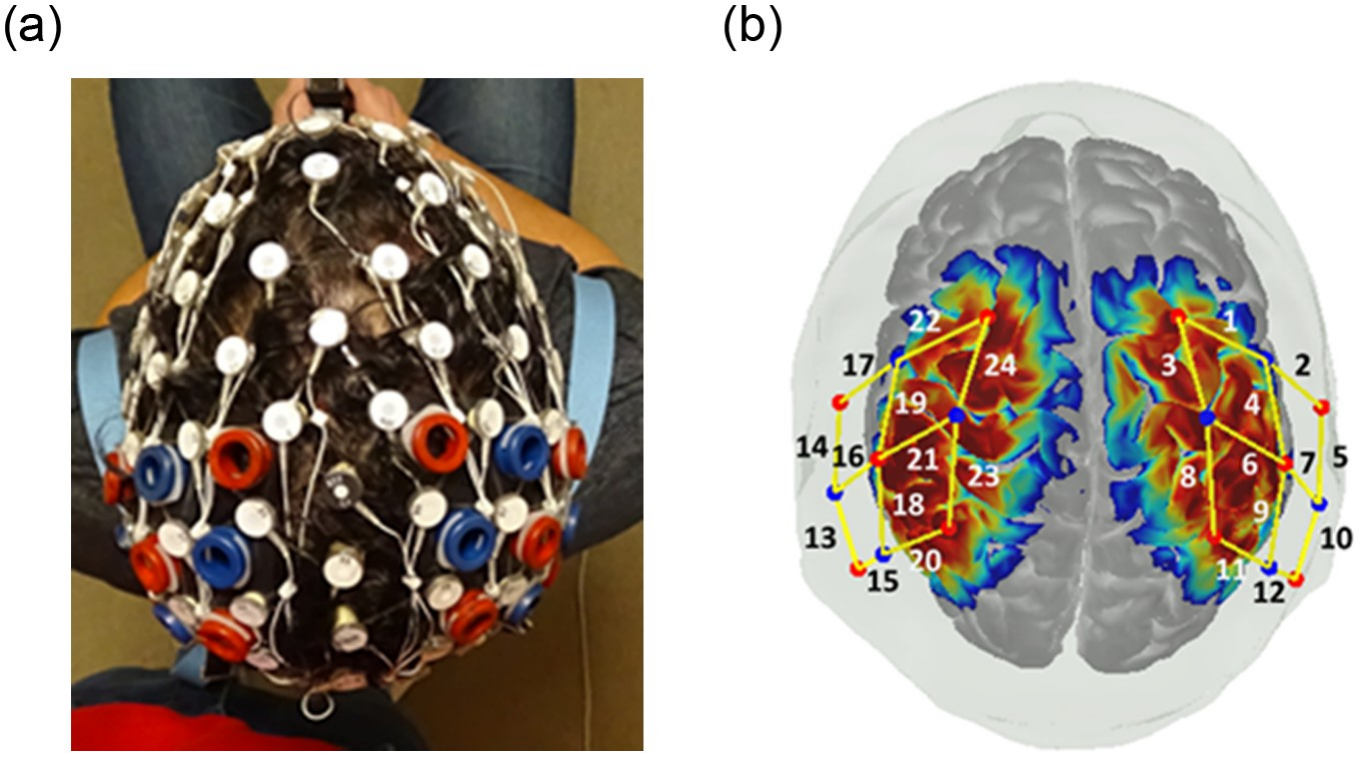
Probe design. (a) fNIRS probe was embedded within an elastic, 128-electrode electroencephalogram (EEG) cap. (b) sensitivity profile for probe geometry and channel number. Sources are represented in red, detectors are represented in blue. Color scale indicates relative sensitivity in log 10 units from -1 (blue) to 1 (red).

Changes in the concentration of oxygenated hemoglobin (HbO) and deoxyhemoglobin (HbR) were measured using a 24-channel continuous-wave functional NIRS system (Hitachi ETG-4100). Infrared light was emitted at 695nm and 830nm wavelengths, with a sampling rate of 10Hz. The 24-channel probe configuration included a customized array of 8 sources and 10 detectors. Optodes were placed over sensorimotor and parietal regions with well-established relationship to the AON [11, 44] As part of a larger study, the fNIRS probe was embedded within an elastic, 128-electrode electroencephalogram (EEG) cap (Electrical Geodesic, Inc., Eugene, OR) (Fig 1). EEG caps of different sizes were utilized based on each participant’s measured head circumference; as a result, there was minor variance in the inter-optode spacing (M = 2.88 ± 0.13 cm, range 2.16–3.26). In addition to measuring head circumference, nasion-to-inion distance was also recorded for proper placement of the EEG-NIRS headgear. To further account for any variations in cap placement, after completion of the experimental paradigm, the positions of sources and detectors on the head were recorded in reference to the nasion, inion, and preauricular landmarks using a 3D-magnetic space digitizer after fNIRS data were collected (Fastrak, Polhemus).

### Procedure

After completing the consent form, all participants underwent a health assessment. As a part of a larger study, participants completed a series of self-report questionnaires and tasks for the assessment of social communication skills, motor ability, and cognitive ability. After the behavioral measures were completed, head measurements (head circumference, nasion to inion, left pre-auricular point to right auricular point) were taken for appropriate cap selection and placement. When placing the cap, the central EEG electrode (Cz) was aligned with the cross-section of the nasion-to-inion midpoint and left-to-right preauricular midpoint distances. All fNIRS optodes were secured within the elastic of the EEG cap using custom-designed silicone fixtures (Fig 1a). Using a small hair pick, comb, and hair gel, any hair underneath the optodes was pushed aside to ensure direct contact with the scalp. Finally, a band of Surgilast elastic net dressing was placed over the top of the cap and optode array to further secure their position.

With the cap appropriately placed and secured, the participant was then seated at arm’s length from a table with an extending tray. The experimenter was positioned on the opposite side of the table facing the participant, with a vertically moving curtain separating the two. The experimental paradigm was explained, and the participant completed a series of practice trials for each condition. All sessions were videotaped via digital recordings for subsequent coding purposes.

After the placement of EEG and fNIRS, the participant and experimenter were seated on opposite sides of a table, facing each other. A stage, similar to a puppet theater, was placed on top of the table to allow for a curtain to be drawn between stimulus presentations and for the tabletop to be pushed out toward participants. A computer screen was placed in the experimenter right-hand side of the stage and out of sight of the participant showing which trial was going to appear before the pre-recorded audio. In addition, it also included information regarding the object (clear cup, red cup, etc). This allowed the experimenter to set up the right object before the curtain went up and the pre-recorded audio instructed which trial was going to happen. The paradigm consisted of an Execute condition (15 trials) and an Observe condition (15 trial). The trial order was randomized, such that no condition type appeared more than three times in sequence. For the execute trials, the participant was instructed to reach a cup placed in the middle of the table, grasp the cup with their palm and fingers, and lift and move the cup towards himself/herself to place it on top of a marked line. At the beginning of the execute trials, a rolling tray on top of the table was pushed forward by the experimenter so that the cup was within arm’s length of the participant. During the observation trials, the participant observed the experimenter perform the same action using their right hand. Each trial was approximately 5 seconds long. Five cups differing in color, size and material were used throughout the experimental paradigm, varying in color, size, and material. The appearance of these cups was randomized across trials.

**Fig 2:**
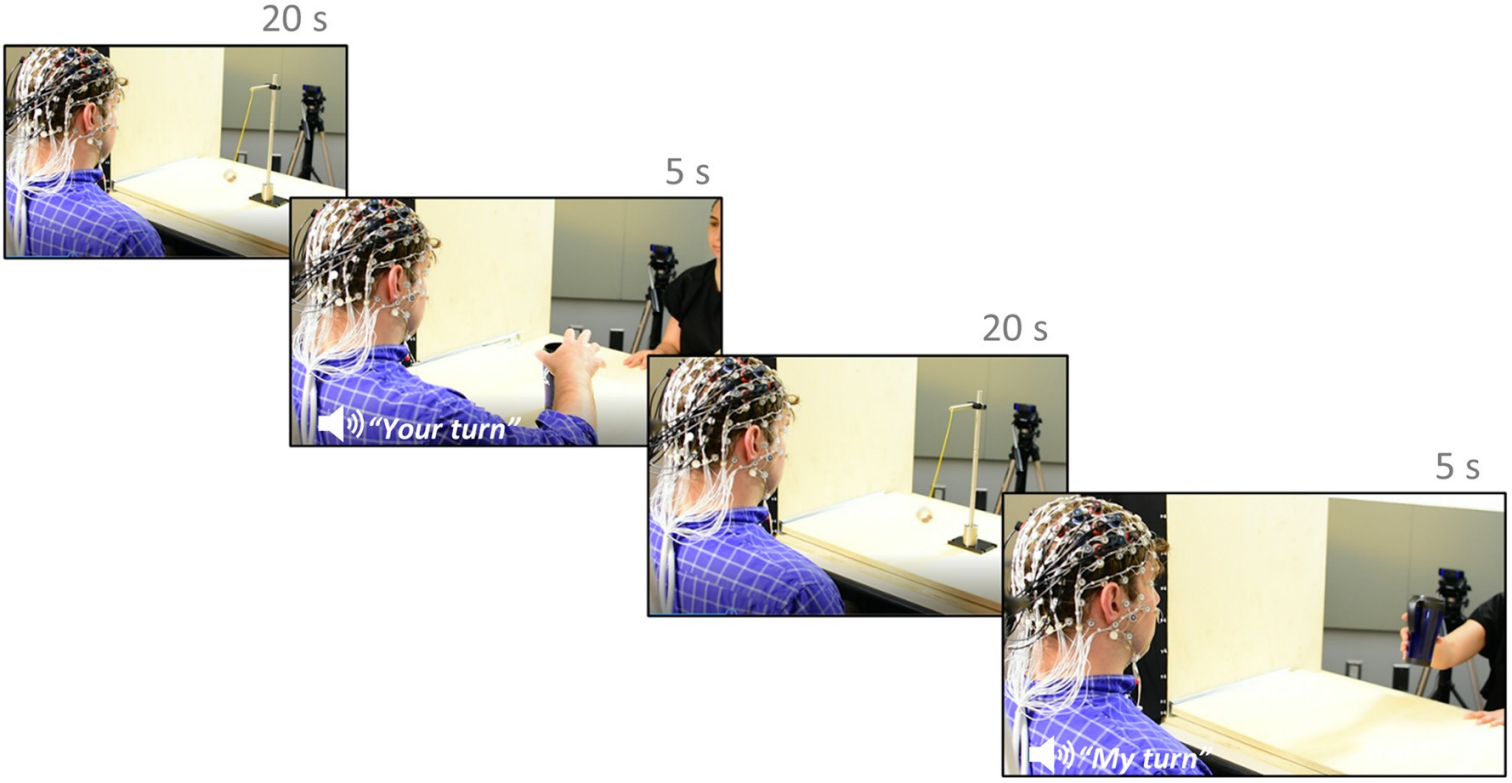
Experimental paradigm. (a and c) Baseline condition (observing a moving pendulum) (b) Action execution (d) Action Observation. The individual in this picture has given written informed consent (as outlined in PLOS consent form) to publish these case details.

Each trial was followed by a 20 second recovery period during which the participant passively looked at a moving pendulum. A moving pendulum (as opposed to a more static stimulus) was chosen with the aim to use this paradigm with young children in future research and compare our results to previous research with young populations that used similar resting conditions [48]. Between each trial and recovery period, a vertically moving curtain was lowered to block the participant’s view as the cup and pendulum were transitioned. The curtain was manually raised once the objects and experimenter were appropriately positioned for the next trial. After raising the curtain at the beginning of each trial, a pre-recorded audio command prompted the task condition. Once the trial was completed, the curtain was lowered, and the object was replaced by the pendulum for the next recovery period (see Fig 2). Altogether, each trial lasted approximately 30 seconds, including 5 seconds of task (i.e. execution, observation), 20 seconds of rest and 5 seconds of task-rest transition when the vertical curtain was lowered to block the participant’s view as the cup was replaced with a pendulum [49]. The experiment took approximately 45 minutes to complete. The participant was given a 5-minute break about halfway through the paradigm.

### Data processing

The video from each session was coded offline using ELAN (version 5.9) to determine the quality of the trials. Trials were considered valid if: 1) the curtain went up and down correctly; 2) the experimenter and the participant did not present early or unnecessary shoulder or arm movement, or movement of the head (e.g., shook their head or fiddled with their fingers; 3) the reaching action occurred after the auditory command in observe trials or after the tray was pushed out for execute trials; 4) the movement was continuous (some participants may have started to reach by moving their shoulder, but had staggered movement before continuing reaching with their arm and hand). We used *start of action* (frame in which the experimenter or participant start moving their shoulder to reach the object) as the marker for observation and execution, respectively. Participants were only included if they completed at least five valid trials of a condition. We established >85% inter-rater reliability for the codes between a primary coder and a secondary coder for 20% of the participants as done in previous studies [50]. After establishing reliability, the remaining dataset was coded by the primary coder. Participants completed an average of 13.5 ± 2.16 valid execution trials (range 5–15) and 13.3 ± 2.43 (6–15) valid observation trials. No statistical difference was found for the number of trials completed between conditions (*χ2* = 34.3, *p* = 0.558).

Changes in concentration of oxy-hemoglobin (HbO) or deoxy-hemoglobin (HbR) measured in micromolar (μm) were processed using HOMER2 (MGH–Martinos Center for Biomedical Imaging, Boston, MA, USA), a MATLAB (The MathWorks, Inc., Natick, MA, USA) software package. After behavioral coding, only valid trials were retained in HOMER2 for data processing. The attenuated light intensities measured by the detecting optodes were converted to optical density units and assessed for movement artifact using Principal Component Analysis (PCA) set at 0.9 [51] PCA was applied to artifactual parts of the signal time-course across all channels by applying an orthogonal transformation to this dataset composed by the total number of channels (24) to produce uncorrelated components ordered by their contribution to the variance of the data. As motion artifacts constitute the largest proportion of variance in the data, removing the components from the data should result in the correction of motion artifacts [52]. Data were low-pass filtered at 0.5 Hz [53] and used to calculate the change in concentration of the hemoglobin chromophores according to the modified Beer-Lambert Law [54] assuming a pathlength factor of 6 for both wavelengths [55]. Traces were segmented into 25-second epochs around the trigger stimulus for each trial (*start action*), with each epoch starting 5 seconds prior to each stimulus. Baseline correction corresponded to the mean HbO/HbR values from -5 to 0 seconds. Epochs include a 5 second period when the curtain was closed. The hemodynamic response function was then generated for each channel during each condition for each participant by averaging the response curves from all trials within a condition into a single hemodynamic curve. For each participant at each channel, the maximum change in HbO (increase in chromophore concentration) and HbR (decrease in chromophore concentration) between 5 and 20 seconds in response to each experimental condition (observation and execution) were computed to be used as the dependent variable in subsequent analyses as in previous fNIRS studies [56, 57] Due to a greater signal to noise ratio and similarly to previous fNIRS studies we only used HbO signal for remaining analysis [58, 59].

#### Atlas viewer

**Fig 3:**
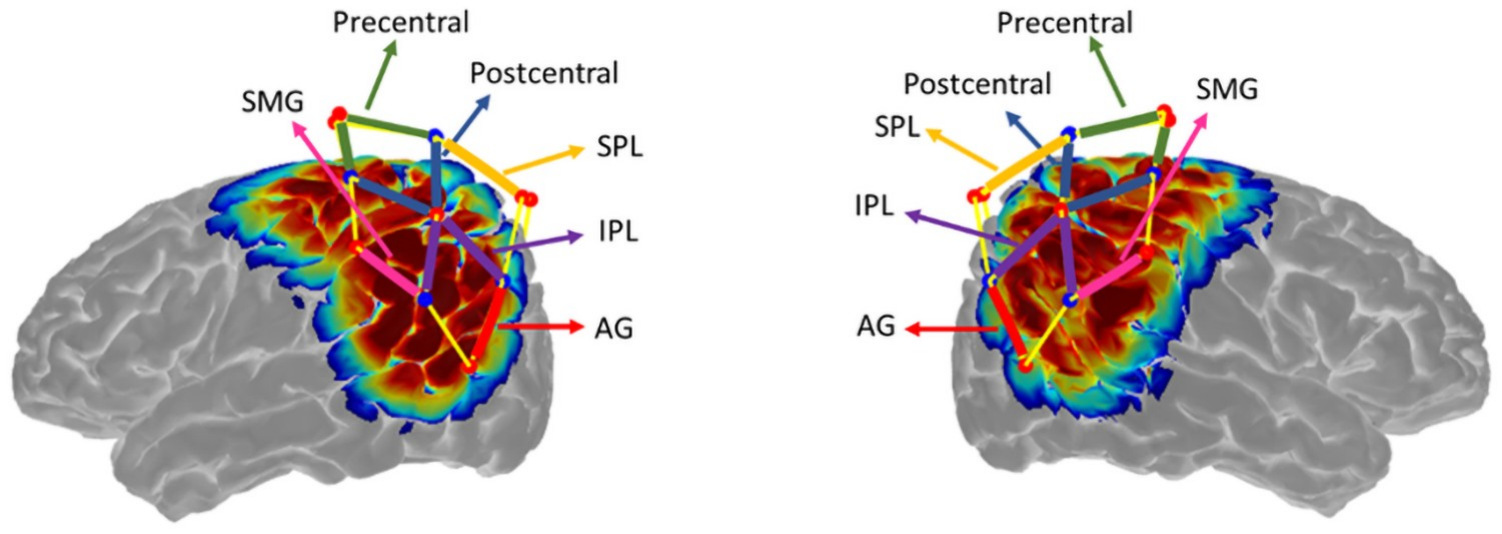
Measurement channels. Six ROIs were derived from the 12 channels located in each hemisphere: pre-central region, post-central region, superior parietal lobule (SPL), inferior parietal lobule (IPL), supra-marginal gyrus (SMG) and angular gyrus (AG).

To determine the anatomical regions covered by each fNIRS channel within each participant, the optode coordinates taken from the Polhemus digitizer were entered into AtlasViewer [60] to scale the Colin brain atlas to each participant’s head. Atlas Viewer generated the MNI coordinates of each channel and the regions of interest (ROI) for each channel was identified. Due to the differences in head size, channels were not consistently positioned over the same brain region for all participants. Hence, the analyses were conducted based on an ROI approach. An ROI was only considered in the analyses if it was covered by at least 50% of participants. When a participant had multiple channels covering one ROI, an average of the maximum HbO amplitude across all the channels for that ROI was generated to be used in subsequent analyses. The six ROI derived from the measurement channels are included in Fig 3.

Statistical analysis was performed using SAS (Statistical Analysis Software) 9.4v. For each ROI, the maximum change in HbO was first assessed relative to the baseline using paired t-tests. Following this initial analysis, a mixed model was computed using the ROIs that showed an increase in HbO hemodynamic activity relative to baseline to examine the contrasts between observation and execution by ROI Reported *p* values are Bonferroni adjusted.

## Results

### Effect of condition against baseline

#### Observation

**Fig 4:**
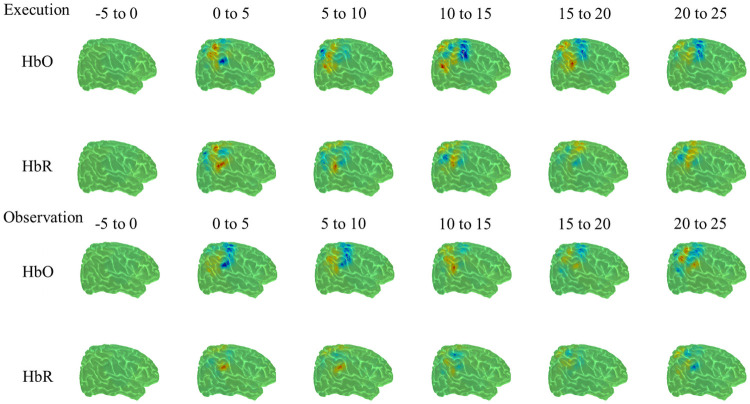
HbO and HbR reconstruction maps for execution and observation in the right hemisphere from -5 to 25 seconds.

**Fig 5:**
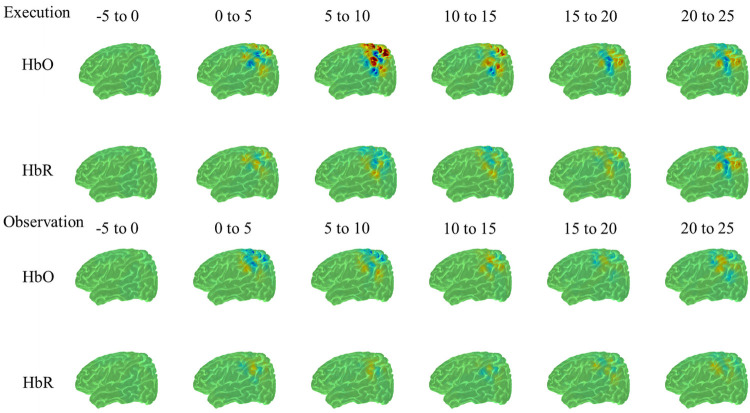
HbO and HbR reconstruction maps for execution and observation in the left hemisphere from -5 to 25 seconds.

The analyses revealed significant hemodynamic increase in HbO over the right postcentral region (*t*(54) = 5.40, *p*<0.001), right precentral region (*t*(50) = 3.8, *p*<0.001), right supra-marginal region (*t*(50) = 4.650, *p*<0.001), right inferior parietal region (*t*(58) = 6.3 *p*<0.001), right superior parietal region (*t*(42) = 4.37, *p*<0.001) and right angular region (*t*(58) = 5.38, *p*<0.001). Significant hemodynamic increase in HbO was also observed in the left supra-marginal region (*t*(50) = 5.84, *p*<0.001), left inferior parietal region (*t*(58) = 6.66, *p*<0.001), left angular region (*t*(52) = 4.59 *p*<0.001), left postcentral region (*t*(56) = 6.30, *p*<0.001), left precentral region (*t*(52) = 4.1, *p*<0.001) and left superior parietal region (*t*(36) = 5.93, *p*<0.001). Observation resulted in an increased hemodynamic function bilaterally in precentral, postcentral, superior parietal, inferior parietal, supramarginal and angular regions. Maximum hemodynamic responses for observation by brain region is included in Tables 1 and 2. Figs 4 and 5 show HbO and HbR image reconstruction of the observation condition in the left and right hemisphere.

**Table 1:** HbO2 response to observation and execution conditions in regions of interest (ROI) in the left hemisphere.

Left Hemisphere								
					Condition > Baseline		EXE > OBS	
ROI	N	Condition	Mean	SE	t-test	p value	t-test	p value
**Postcentral_L**	30	Observation	0.082	0.0131	6.300	**<0.0001**	2.98	**0.0058**
		Execution	0.1585	0.0253	6.250	**<0.0001**		
**Precentral_L**	28	Observation	0.074	0.0183	4.100	**0.0010**	3.40	**0.0022**
		Execution	0.2431	0.0491	4.960	**<0.0001**		
**SupraMarginal_L**	27	Observation	0.1101	0.019	5.840	**<0.0001**	0.07	0.948
		Execution	0.1132	0.051	2.200	0.0329		
**Parietal_Inf_L**	30	Observation	0.0901	0.018	6.660	**<0.0001**	1.58	0.1258
		Execution	0.136	0.031	4.340	**<0.0001**		
**Parietal_Sup_L**	20	Observation	0.1254	0.022	5.930	**<0.0001**	0.80	0.4348
		Execution	0.1513	0.038	3.970	**0.0001**		
**Angular_L**	17	Observation	0.124	0.027	4.590	**<0.0001**	0.60	0.5511
		Execution	0.154	0.0502	3.600	0.0035		

Note. Significant values after Bonferroni correction (*p*<0.002) are in bold.

**Table 2:** HbO2 response to observation and execution conditions in regions of interest (ROI) in the right hemisphere.

Right Hemisphere								
					Condition > Baseline		EXE > OBS	
	N	Condition	Mean	SE	t-test	p value	t-test	p value
**Post_Central Right**	29	Observation	0.078	0.015	5.040	**<0.0001**	0.71	0.4841
		Execution	0.092	0.031	2.950	0.0046		
**Precentral_R**	26	Observation	0.062	0.016	3.800	**0.0004**	1.03	0.3149
		Execution	0.091	0.029	3.110	0.0031		
**SupraMarginal_R**	27	Observation	0.078	0.016	4.650	**<0.0001**	1.27	0.2168
		Execution	0.1126	0.031	3.570	**0.0008**		
**Parietal_Inf_Right**	30	Observation	0.094	0.014	6.320	**<0.0001**	1.01	0.3221
		Execution	0.117	0.027	4.400	**<0.0001**		
**Parietal_Sup_Right**	23	Observation	0.095	0.021	4.370	**<0.0001**	1.36	0.1873
		Execution	0.127	0.02	6.320	**<0.0001**		
**Angular_R**	30	Observation	0.086	0.016	5.380	**<0.0001**	0.79	0.4342
		Execution	0.105	0.028	3.730	**0.0004**		

Note. Significant values after Bonferroni correction (*p*<0.002) are in bold.

#### Execution

The analyses revealed a significant hemodynamic increase in HbO over the right supra-marginal region (*t*(50) = 3.57, *p*<0.001), right inferior parietal region (*t*(58) = 4.40, *p*<0.001), right superior parietal region (*t*(42) = 6.32, *p*<0.001) and right angular region (*t*(58) = 3.7, *p*<0.001). Significant hemodynamic increase in HbO was also observed in the left inferior parietal region (*t*(58) = 4.34, *p*<0.001), left postcentral region (*t*(56) = 6.25, *p*<0.001), left precentral region (*t*(52) = 4.96, *p*<0.001) and left superior parietal region (*t*(36) = 3.97, *p*<0.001). Execution resulted in an increased hemodynamic response in bilateral superior and inferior parietal regions. In addition, precentral and postcentral, and angular and supramarginal regions were activated unilaterally, in the left and right hemisphere, respectively. Maximum hemodynamic responses for execution by brain region is illustrated in Tables 1 and 2. Figs 4 and 5 show HbO and HbR image reconstruction of execution condition in the left and right hemisphere.

#### Contrast between conditions

**Fig 6:**
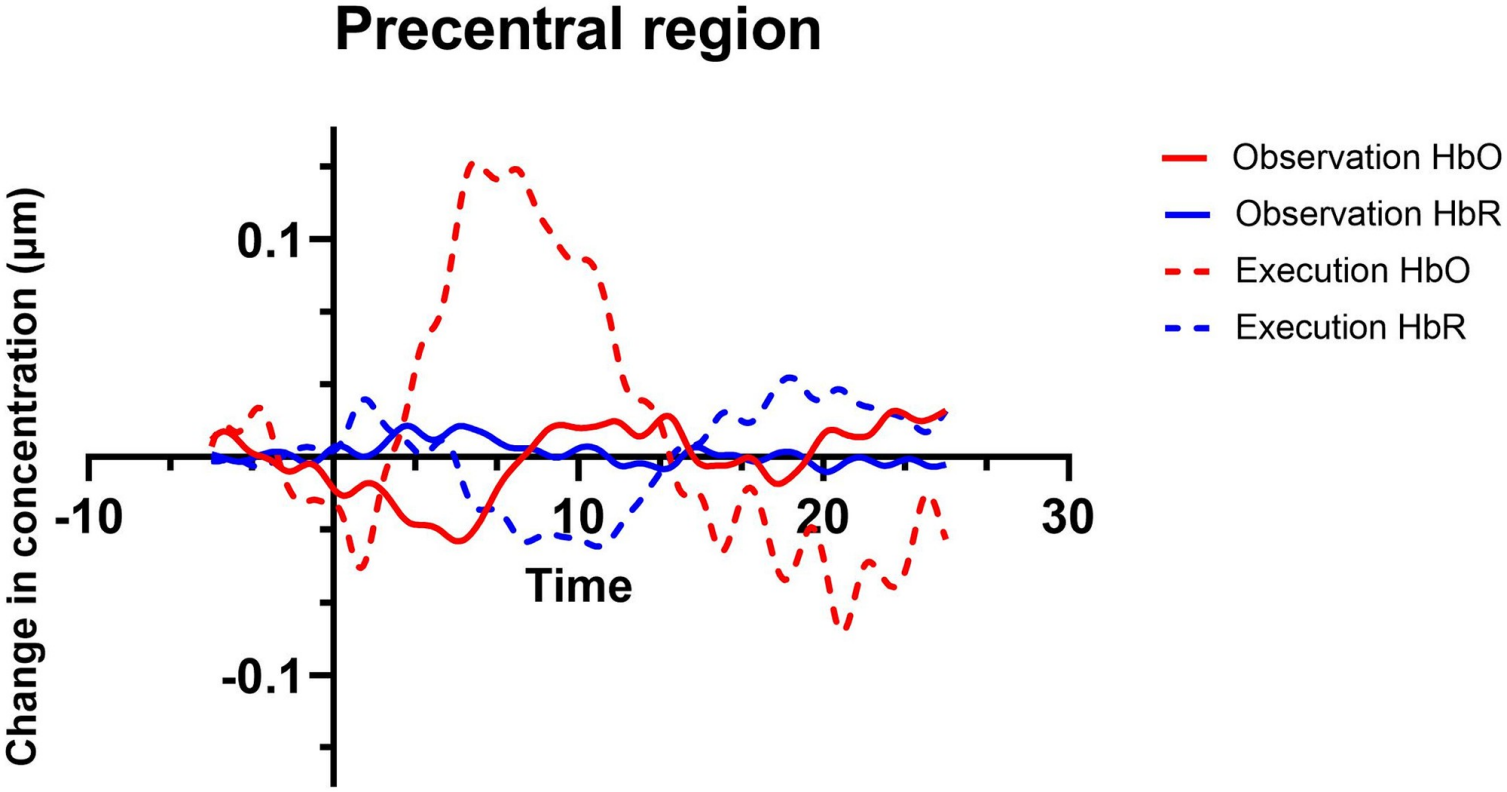
Hemodynamic response function for precentral region during execution and observation conditions.

**Fig 7:**
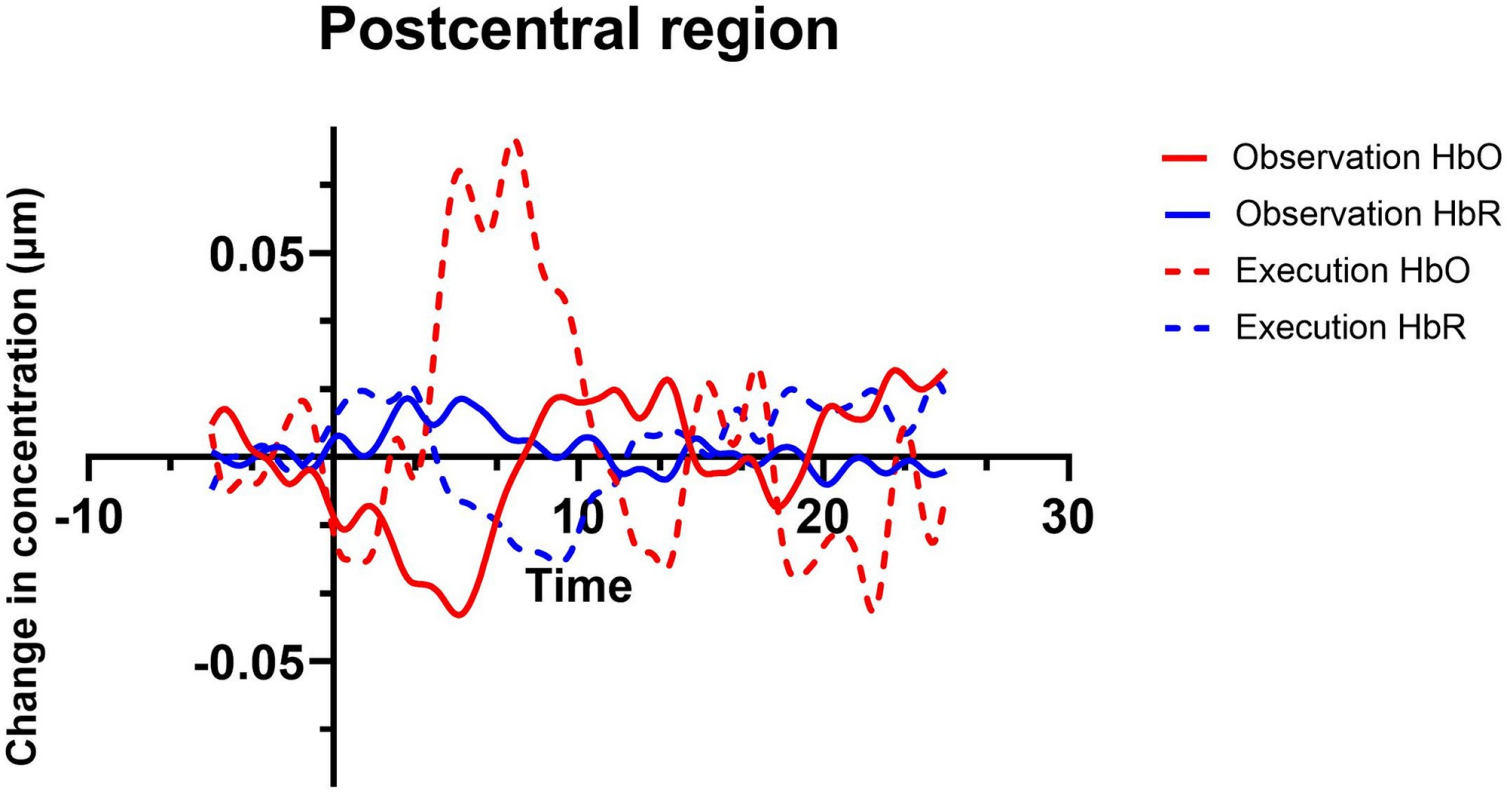
Hemodynamic response function for postcentral region during execution and observation conditions.

For the execution > observation contrast, a significant difference was found over the left precentral region (*M* = 0.1684, *SE* = 0.049; *t*(26) = 3.40, *p*<0.002) and left postcentral region (*M* = 0.2431, *SE* = 0.0491, *t*(28) = 2.98, *p* = 0.0022). See Figs 6 and 7 for the hemodynamic response function on precentral and postcentral region. No significant differences were found for observation > execution contrast. Regions that were activated in both observation and execution conditions include bilateral superior parietal, bilateral inferior parietal, right supramarginal and right angular regions. See Tables 1 and 2 for region-by-region HbO contrasts.

## Discussion

The study of the AON in human adults has traditionally been investigated through paradigms that lack ecological validity. Few studies have characterized brain activity during live paradigms using basic actions like reaching. In this study, we examined the neural correlates of the AON during a live paradigm. Specifically, we used fNIRS to measure brain activity in sensorimotor and parietal regions during execution and observation of an action. Overall, our results confirm previous findings observed in the fMRI literature pointing to bilateral parietal regions as candidate regions of the human *AON*.

Our paradigm elicited brain activation for execution and observation of a simple action. Brain activation for execution was greater in contralateral sensorimotor regions (pre- and post-central regions), similar to previous fNIRS studies [29, 37, 41]. Pre-and post central regions include the primary sensory cortex and somatosensory cortices, respectively, which are involved when performing and learning motor tasks [61, 62]. In addition, the execution condition also elicited activation in bilateral parietal regions, namely the IPL and SPL. The results are not surprising since the parietal cortex encodes different goal-directed actions, such as grasping [63] The SPL for example is believed to be involved in online monitoring/adjusting of the ongoing reaching/grasping action [63, 64], whereas the IPL (including the SMG and AG) appears to be more involved with functional use of objects and tools [65]. Evidence from perturbation studies using transcranial magnetic stimulation to anterior intraparietal sulcus and superior parietal lobule have shown that these brain regions are involved in the integration of on-line changes of a motor plan [63].

In our study the observation condition elicited bilateral activation in sensorimotor regions, IPL, SPL, SMG, and AG. Similarly results have been found in previous fNIRS [30, 31] and fMRI studies [5, 6]. Observing an action is known to elicit activation of bilateral networks with involvement of frontal (premotor and primary motor) and parietal regions [6]. Interestingly, observation of an action is more associated with activity in the inferior/superior parietal cortex compared with other conditions, namely imagining an action [61]. Another finding that goes along previous studies is the lower mean amplitude of observation compared with execution [30]. In our study, we did not conduct analysis to test differences between ROIs within a condition, but this could be addressed in future studies.

One of the main questions regarding AON that remains to be answered is what brain regions overlap between observation and execution conditions. In the current study, the brain regions that shared activation between conditions included the bilateral IPL, bilateral SPL, right SMG and RA as indicated by significantly higher HbO than baseline in both the observe and execute conditions. Previous fNIRS studies have found parietal regions active during observation and execution conditions [29, 32, 33, 41, 66]. Nevertheless, most studies covered only ROIs in the left hemisphere which limits the conclusions that can be drawn. In addition, few studies conducted contrasts between conditions to determine if the effects between the two were statistically different (or similar) [35, 36]. Although all regions covered by the fNIRS probe were shown to be active in the observation and execution conditions when activation was compared to baseline, contrasts between the two conditions revealed no difference in activation in left parietal and right hemisphere regions, indicating that these regions may contribute to the AON. fMRI studies have also found shared activation in these regions [6, 21], which provides stronger evidence that parietal cortex might play an important role in the AON in the human brain, similarly to the homologue PF/PFG in the macaque monkey [67].

Another brain region that has been implicated in the human and non-human primate brain mirroring system is the vPMC [5–7, 21]. Our probe was limited to sensory motor and parietal regions, which is a limitation of the present study. Considering the putative role of frontal networks on the mirroring system [68], it is likely that PMC is also involved in the AON and future studies should include all three brain regions.

Our study contributes to a better understanding of the mirroring properties of the human brain through a well-controlled live-action paradigm. However, a few limitations should be considered when interpreting the presented findings. Although most of our participants were right-handed, a few participants were ambidextrous. All of them reported that they reached/grasped with their right hand in everyday activities and they all reached using their right hand throughout the paradigm. Another limitation is the limited number of optodes of our fNIRS system, which limited the ROIs examined. We were not able to cover, for example, frontal regions that have been involved in the human AON [5]. Future studies examining the AON using fNIRS should include a probe that covers frontal (vPMC), sensorimotor (S1 and M1 as control regions) and parietal regions (SPL and IPL) bilaterally. This would allow to contrast between regions and conditions, resulting in a full depiction of the human AON. Finally, given the “live” or naturalistic design of the paradigm, the execution condition in the study involved participants observing their own hand performing the reach and grasp action. Therefore, responses during the execute trials likely do not reflect pure motor responses and involve observation, and thus the shared brain activation across conditions may be driven by participants’ observation of the motion.

### Future directions

Further exploration of activation during a live AON paradigm in healthy adults is warranted. Although a number of studies have examined activation across various cortical regions during such tasks, other analytical methods could reveal more information about the function on this network. Specifically, follow up connectivity analyses would be useful to better understand the AON and determine more specifically how these regions are interconnected. Due to its proposed role in complex behaviors, such as social-communicative deficits, it is possible that additional detail about the structures involved in these processes would be revealed using a more network-driven analysis.

Not only is fNIRS appropriate to use during naturalistic settings like social interaction and AON paradigms, its portability and affordability allow for the study of AON longitudinally. fNIRS is gaining notoriety in the developmental literature for use with infants and toddlers due to these features [69] making it a favorable methodology for use in a longitudinal framework. This would be critical for 1) understanding the developmental trajectory of the AON; 2) understanding the AON in populations that lack social reciprocity, namely infants and children at risk of ASD. While EEG has largely been used to study the AON in infant and child populations, the present study shows evidence that fNIRS is a viable alternative for studies probing this network and may minimize concerns with motion artifact and spatial resolution that are often cited in the developmental literature. A recent pilot study has used fNIRS to examine differences in cortical activation patterns to action observation, action execution and interpersonal synchrony in children with ASD and has found a distinct response, including hyper-activation in the IPL compared with controls, in addition to brain-behavior relationships [38]. These preliminary findings support the use of the AON to study of younger populations using a simple motor paradigm like the one we are proposing or a paradigm that comprises simple and complex motor actions, which can be also administered to examine distinct cortical patterns using fNIRS [70].

In conclusion, this study investigates the neural correlates of AON using fNIRS and a well-controlled paradigm including observation and execution of an action. The findings suggest that *mirroring properties* of the human brain might be localized in the parietal cortex, more specifically in IPL (including AG and SMG). The findings highlight the applicability of fNIRS for the study of AON, which can offer valuable insight to its function on neurodevelopmental disorders.
